# Role of Prostaglandins in Nitric Oxide-Induced Glial Cell-Mediated Vasodilation in Rat Retina

**DOI:** 10.3390/biom12101403

**Published:** 2022-10-01

**Authors:** Asami Mori, Haruka Seki, Satoru Mizukoshi, Takashi Uezono, Kenji Sakamoto

**Affiliations:** Laboratory of Medical Pharmacology, Department of Clinical & Pharmaceutical Sciences, Faculty of Pharma-Sciences, Teikyo University, 2-11-1 Kaga, Itabashi-ku, Tokyo 173-8605, Japan

**Keywords:** glial cell, nitric oxide, prostanoid EP_2_ receptor, retina

## Abstract

We previously identified that NO derived from neuronal cells acts on glial cells and causes vasodilation in the healthy rat retina via the release of epoxyeicosatrienoic acids (EETs) and prostaglandins (PGs) by activation of the arachidonic acid cascade. However, it is not clear which PG types are involved in these responses. The aim of the present study was to identify prostanoid receptors involved in glial cell-derived vasodilation induced by NO in rat retina. Male Wistar rats were used to examine the effects of intravitreal pretreatment with indomethacin, a cyclooxygenase inhibitor; PF-04418948, a prostanoid EP_2_ receptor antagonist; and CAY10441, a prostanoid IP receptor antagonist, on the changes in the retinal arteriolar diameter induced by intravitreal administration of NOR3, an NO donor. Retinal arteriolar diameters were measured using ocular fundus images captured with a high-resolution digital camera in vivo. The increase in the retinal arteriolar diameter induced by intravitreal injection of NOR3 was significantly suppressed by intravitreal pretreatment with indomethacin and PF-04418948, but not by CAY10441. The dose of PF-04418948 and CAY10441 injected intravitreally in the present study significantly reduced the increase in the retinal arteriolar diameter induced by prostaglandin E_2_ (PGE_2_) and prostaglandin I_2_ (PGI_2_), respectively. These results suggest that activation of the arachidonic acid cascade and subsequent stimulation of prostanoid EP_2_ receptors are involved in rat retinal vasodilatory responses evoked by NO-induced glial cell stimulation. Therefore, glial cell-derived PGE_2_, similar to EETs, may play an important role in retinal vasodilatory mechanisms.

## 1. Introduction

In the central nervous system, the concept of the neurovascular unit, that is, neuronal, glial, and vascular cells, functioning together in the homeostatic hemodynamic response, has been proposed [1]. In the brain, stimulation of neurons has been reported to induce a dilation response of arterioles mediated by glial cells [2]. In the retina, a part of the central nervous system, the neurovascular unit is organized in the same manner as in the brain [3], and retinal neuronal activity is an important factor in the regulation of retinal blood flow [4].

Recently, we reported that NO derived from neuronal NO synthase (nNOS) acts on glial cells to dilate arterioles in the healthy rat retina by stimulating *N*-methyl-D-aspartic acid (NMDA) receptors [5]. We have also reported that intravitreal administration of NOR3, an NO donor, causes a glial cell-dependent retinal vasodilatory response rather than a direct effect on the retinal arterioles in rats [5,6]. This indicates that intravitreal injection of the NO donor can provide a vasodilatory response that is mainly mediated by glial cell stimulation. Furthermore, we found that NO in the vitreous causes retinal vasodilation via the release of glial cell-derived epoxyeicosatrienoic acids (EETs) and vasodilatory prostaglandins (PGs). These previous studies suggest that arachidonic acid cascade metabolites are involved in vasodilation via neuronal or glial cell stimulation in the retina.

Vasodilatory PGs are important for hemodynamic regulation of the retina [4]. We previously reported the effects of vasodilatory PGs on the diameters of rat retinal vessels in literature. Intravenous infusion of prostaglandin I_2_ (PGI_2_) and prostaglandin E_2_ (PGE_2_) markedly dilates rat retinal arterioles. In addition, stimulation of the prostanoid EP_2_ receptor, one of the receptors on which PGE_2_ acts in a dilatory manner, dilates the retinal arterioles [7]. Although NO dilates blood vessels via activation of guanylyl cyclase, the expression of guanylyl cyclase in rat retinal blood vessels is low and the NO-induced vasodilation response is cyclooxygenase-dependent [8]. Intravenous infusion of NO donors induces dilation of retinal arterioles via PGI_2_ production and release [9]. Thus, our previous studies have indicated that PGI_2_ and/or PGE_2_ are important in the regulation of vascular tone in the retina. Our recent studies on neurovascular units in the retina have shown that vasodilatory PGs are involved in the glial cell-stimulated dilation of retinal arterioles. However, the types of PGs that are produced and released from glial cells remain unclear. The present study aimed to identify prostanoid receptors involved in NO-induced glial cell-derived vasodilation in rat retina.

## 2. Materials and Methods

### 2.1. Animals

The experimental procedures conformed to the Regulations for the Care and Use of Laboratory Animals adopted by the Animal Care and Use Committee of Teikyo University (approval number: 20-013).

Forty-four male Wistar rats (7–9 weeks old, Japan SLC Inc., Hamamatsu, Japan) were housed at 24 °C, 50% humidity, and 12-h light–dark cycle with free access to standard rat chow (CRF-1, Oriental Yeast Co., Ltd., Tokyo, Japan) and tap water.

### 2.2. Reagents

The following reagents were used: 4,5-dihydro-N-[4-[[4-(1-methylethoxy)phenyl]methyl]phenyl]-1H-imadazol-2-amine (CAY10441), 1-(4-fluorobenzoyl)-3-[[(6-methoxy-2-naphthalenyl)oxy]methyl]-3-azetidinecarboxylic acid (PF-04418948), prostaglandin E_2_ (PGE_2_), prostaglandin I_2_ (PGI_2_) (Cayman Chemical Co., Ann Arbor, MI, USA), (±)-(E)-4-ethyl-2-[(E)-hydroxyimino]-5-nitro-3-hexenamide (NOR3; Dojindo, Kumamoto, Japan), dimethyl sulfoxide, tetrodotoxin (Nacalai Tesque, Kyoto, Japan), fluorescein sodium salt, indomethacin, methoxamine hydrochloride (Sigma-Aldrich, St. Louis, MO, USA), pontamine sky blue 6B, pentobarbital sodium (Tokyo Chemical Industry, Tokyo, Japan), and hydroxyethyl cellulose (Scopisol 15^®^; Senju Pharmaceutical, Osaka, Japan).

### 2.3. Surgical Protocols

A slightly modified version of our previously described surgical protocols [5,9] was used. The rats were anesthetized by intraperitoneal injection of pentobarbital sodium (50 mg/kg). Catheters were inserted into the jugular and femoral veins for drug administration and into the femoral artery for measuring blood pressure and heart rate. Blood pressure and heart rate were monitored and recorded using PowerLab (AD Instruments, Bella Vista, Sydney, Australia). The trachea was cannulated, and the animals were mechanically ventilated. To capture fundus images at the same angle throughout the experiment, eye movements were blocked by treating rats with intravenous (i.v.) injection of tetrodotoxin (50 µg/kg). Treatment with tetrodotoxin reduces systemic blood pressure; therefore, methoxamine hydrochloride (15–40 µg/kg/min, i.v.) was continuously infused to maintain adequate systemic circulation. Additional pentobarbital sodium (10 mg/kg) was administered, as required.

### 2.4. Fundus Photography and Measurement of the Retinal Arteriole Diameter

Fundus photography and measurement of retinal arteriole diameter were performed as described previously [5,9]. Fundus photographs were taken with a digital camera (EOS7D; Canon, Tokyo, Japan) and borescope-type objective lens (Model 01; Scalar, Tokyo, Japan). The region including the retinal arteriole (138 × 276 pixels, 138 × 276 µm) was clipped from the original fundus photograph (5184 × 3456 pixels, 5184 × 3456 µm) to measure the diameter of the retinal arteriole.

### 2.5. Experimental Protocols

#### 2.5.1. Protocol 1: Role of Prostaglandins in Retinal Vasodilator Responses to NOR3

We examined the effects of indomethacin (10 nmol/eye), a cyclooxygenase inhibitor, and vehicle (10% dimethyl sulfoxide in saline) on the changes in retinal arterioles, blood pressure, and heart rate induced by intravitreal injection of NOR3 (5 nmol/eye). Indomethacin or vehicle was administered via intravitreal injection before the surgery. The indomethacin dose was chosen based on our previous study [8].

#### 2.5.2. Protocol 2: Role of Prostanoid EP_2_ Receptor in Retinal Vasodilator Responses to NOR3

Next, we examined the possible involvement of the prostanoid EP_2_ receptor in NOR3-induced vasodilation in the retina. PF-04418948 (20 nmol/eye), a prostanoid EP_2_ receptor antagonist, or vehicle (50% dimethyl sulfoxide in saline) was administered by intravitreal injection before the surgery. The dose of PF-04418948 was determined by evaluating the dilation of the retinal arterioles by intravenous infusion of PGE_2_ (0.1–30 µg/kg/min). PGE_2_ was dissolved in ethanol and diluted with saline (final concentration of ethanol was 0.9%).

#### 2.5.3. Protocol 3: Role of Prostanoid IP Receptor in Retinal Vasodilator Responses to NOR3

To examine the possible involvement of the prostanoid IP receptor in NOR3-induced vasodilation in the retina, CAY10441 (10 nmol/eye), a prostanoid IP receptor antagonist, or vehicle (50% dimethyl sulfoxide in saline) was administered by intravitreal injection before surgery. The dose of CAY10441 was determined by evaluating the inhibitory effect of CAY10441 on retinal arteriole dilation by intravenous infusion of PGI_2_ (0.03–10 µg/kg/min). PGI_2_ was dissolved in 0.05 M glycine-NaOH buffer (pH 9.0) [9].

### 2.6. Data Analysis

The retinal arteriolar diameter, mean arterial pressure, and heart rate were expressed as percentages (%) of the baseline level (mean values of the data obtained from time −2 to 0 min). The integrated area under the NOR3-induced vasodilator response curve (AUC) was calculated for each rat. The mean value of control (vehicle-treated) rats was normalized to 100%. All values are presented as the mean ± standard error (S.E.M.). An unpaired t-test was used to compare the baseline values and the area under the NOR3-induced response curve values between the two groups. When comparing responses to NOR3, PGE_2_, or PGI_2_, two-way ANOVA followed by the Tukey post-hoc test was used (PRISM6, GraphPad Software, San Diego, CA, USA). Differences were considered statistically significant when the *p*-value was less than 0.05.

## 3. Results

### 3.1. The Baseline Value of Retinal Arteriolar Diameter, Mean Arterial Pressure, and Heart Rate

The baseline values for retinal arteriolar diameter, mean arterial pressure, and heart rate for each protocol are shown in Table 1. There were no differences in the baseline values between any of the groups.

### 3.2. Role of Prostaglandins in Retinal Vasodilator Responses to NOR3

**Figure 1:**
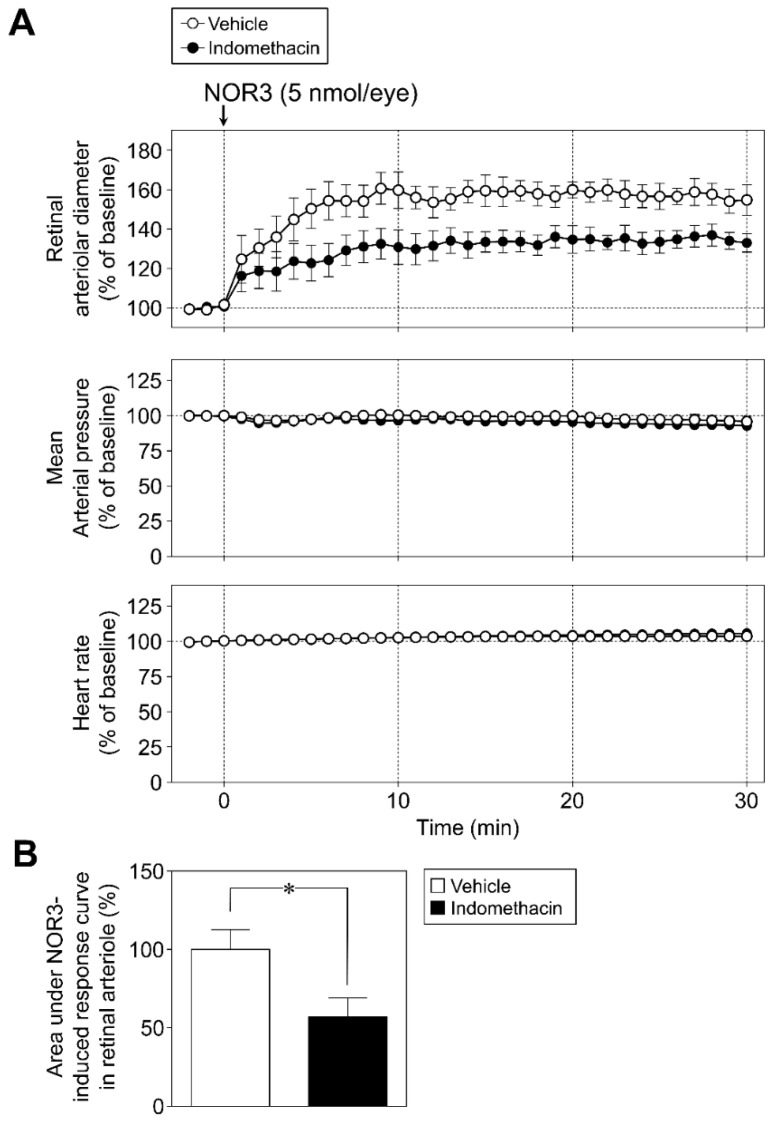
Involvement of prostaglandins in intravitreal injection of NOR3-induced retinal arteriolar dilation in rats. (**A**): The upper, middle, and bottom panels show the effect of indomethacin (10 nmol/eye), a cyclooxygenase inhibitor, on changes in retinal arteriolar diameter, mean arterial pressure, and heart rate induced by intravitreal injection of NOR3 (5 nmol/eye), respectively. (**B**): Bar graph shows the integrated area under the NOR3-induced response curve in retinal arteriole in vehicle- and indomethacin-treated groups. Each point or column with a vertical bar represents the mean ± S.E.M. from five animals. * *p* < 0.05.

The intravitreal injection of NOR3 (5 nmol/eye) increased the retinal arteriolar diameter without changing the mean arterial pressure and heart rate (Figure 1A). Intravitreal injection of indomethacin attenuated the NOR3-induced increase in retinal arteriolar diameter response without affecting mean arterial pressure and heart rate (Figure 1A). Analysis of the area under the NOR3-induced response curve in retinal arterioles confirmed the significant inhibitory effect of indomethacin on NOR3-induced responses (Figure 1B).

### 3.3. Role of Prostanoid EP_2_ Receptor in Retinal Vasodilator Responses to NOR3

**Figure 2:**
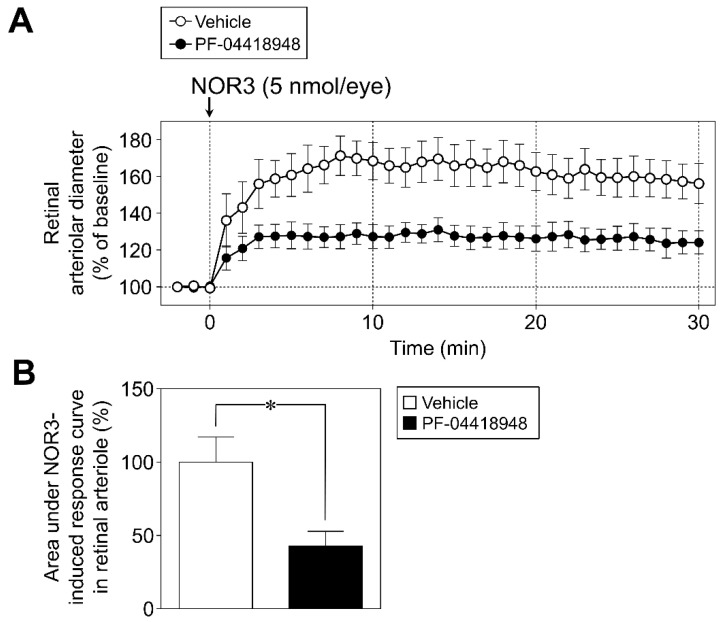
Involvement of prostanoid EP_2_ receptors in intravitreal injection of NOR3-induced retinal arteriolar dilation in rats. (**A**): The effect of PF-04418948 (20 nmol/eye), a prostanoid EP_2_ receptor antagonist, on changes in retinal arteriolar diameter induced by intravitreal injection of NOR3 (5 nmol/eye). (**B**): Bar graph shows the integrated area under the NOR3-induced response curve in retinal arteriole in vehicle- and PF-04418948-treated groups. Each point or column with a vertical bar represents the mean ± S.E.M. from five to six animals. * *p* < 0.05.

**Figure 3:**
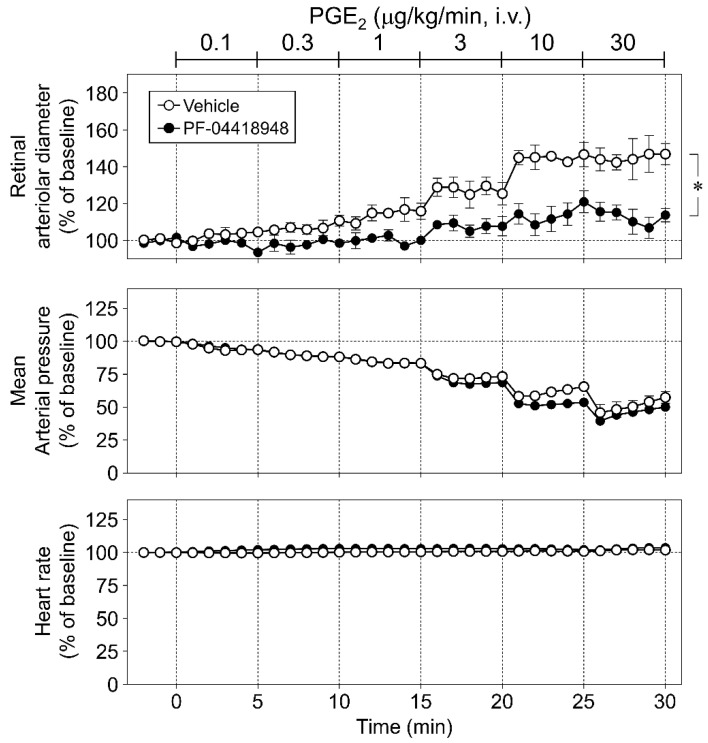
Involvement of prostanoid EP_2_ receptors in intravenous infusion of prostaglandin E_2_ (PGE_2_)-induced retinal arteriolar dilation in rats. The upper, middle, and bottom panels show the effect of PF-04418948 (20 nmol/eye) on changes in retinal arteriolar diameter, mean arterial pressure, and heart rate induced by intravenous infusion of PGE_2_ (0.1–30 µg/kg/min), respectively. Each point with a vertical bar represents the mean ± S.E.M. from three animals. * *p* < 0.05.

We examined the effects of PF-04418948 (20 nmol/eye) on PGE_2_- and NOR3-induced responses. The increase in retinal arterial pressure following intravitreal injection of NOR3 (5 nmol/eye) tended to be suppressed by PF-04418948 (Figure 2A). Analysis of the area under the NOR3-induced response curve in the retinal arterioles indicated that the inhibitory effect was significant (Figure 2B). PGE_2_ (0.1–30 µg/kg/min, i.v.)-induced vasodilator responses in the retinal arterioles were markedly inhibited by intravitreal injection of PF-04418948 (20 nmol/eye). In contrast, PF-04418948 had no effect on PGE_2_-induced changes in blood pressure or heart rate (Figure 3).

### 3.4. Role of Prostanoid IP Receptor in Retinal Vasodilator Responses to NOR3

**Figure 4:**
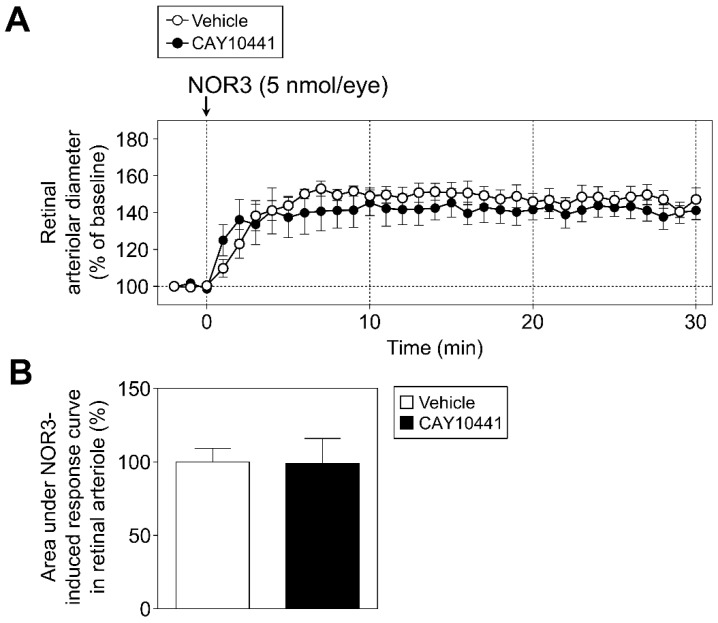
Involvement of prostanoid IP receptors in intravitreal injection of NOR3-induced retinal arteriolar dilation in rats. (**A**): The effect of CAY10441 (10 nmol/eye), a prostanoid IP receptor antagonist, on changes in retinal arteriolar diameter induced by intravitreal injection of NOR3 (5 nmol/eye). (**B**): Bar graph shows the integrated area under the NOR3-induced response curve in retinal arteriole in vehicle- and CAY10441-treated groups. Each point or column with a vertical bar represents the mean ± S.E.M. from four to five animals.

**Figure 5:**
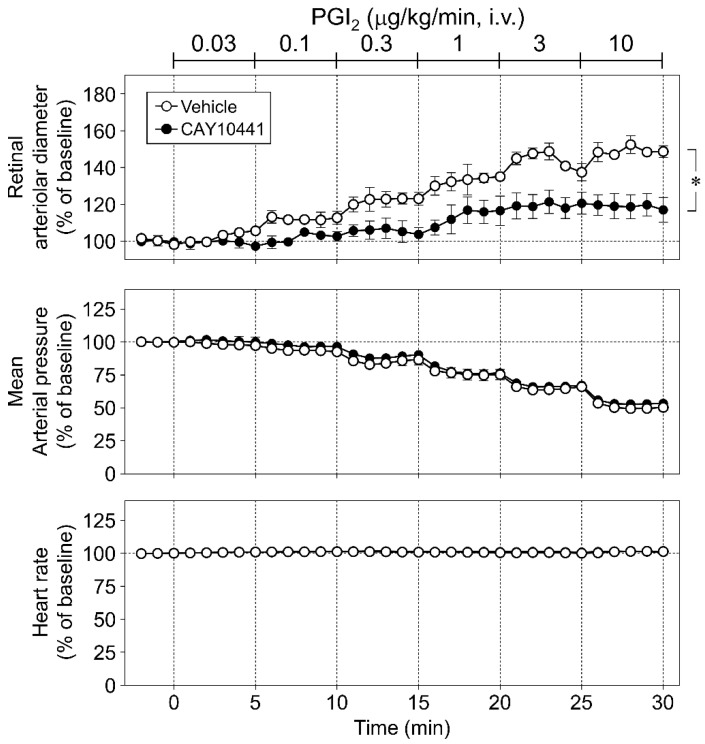
Involvement of prostanoid IP receptors in intravenous infusion of prostaglandin I_2_ (PGI_2_)-induced retinal arteriolar dilation in rats. The upper, middle, and bottom panels show the effect of CAY10441 (10 nmol/eye) on changes in retinal arteriolar diameter, mean arterial pressure, and heart rate induced by intravenous infusion of PGI_2_ (0.03–10 µg/kg/min), respectively. Each point with a vertical bar represents the mean ± S.E.M. from four animals. * *p* < 0.05.

Next, we examined the effects of CAY10441 (10 nmol/eye) on the PGI_2_- and NOR3-induced responses. The retinal vasodilatory response to NOR3 (5 nmol/eye) was unaffected by CAY10441 (Figure 4A,B). However, this dose of CAY10441 (10 nmol/eye) significantly inhibited PGI_2_ (0.03–10 µg/kg/min, i.v.)-induced retinal vasodilatory responses without affecting blood pressure and heart rate (Figure 5). Increasing the dose of CAY10441 from 10 nmol/eye to 50 nmol/eye evoked no further inhibitory effect, and the changes in retinal arteriolar diameter induced by PGI_2_ (10 µg/kg/min, i.v.) were 48.9 ± 2.7% (*n* = 4) in the vehicle group, 18.8 ± 6.3% (*n* = 4) in the CAY10441 (10 nmol/eye) group, and 26.5 ± 4.2% (*n* = 4) in the CAY10441 (50 nmol/eye) group.

## 4. Discussion

**Figure 6:**
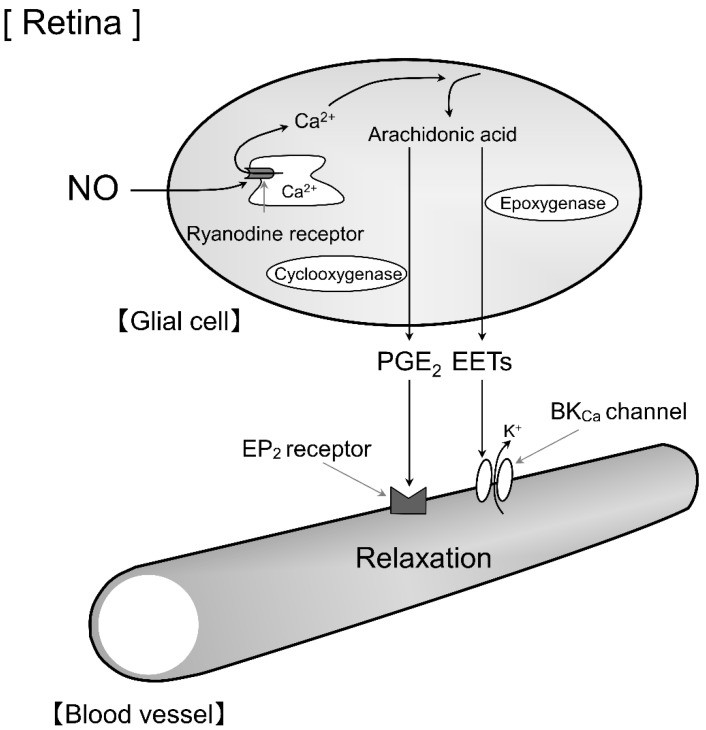
Schema of glial cell-mediated vasodilation mechanisms in the retina. Stimulation of NO on glial cells enhances ryanodine receptor-mediated Ca^2+^ release and thereby stimulates production of prostaglandin E_2_ (PGE_2_) or epoxyeicosatrienoic acids (EETs) in the cells. The released PGE_2_ and EETs dilate retinal blood vessels through stimulation of the prostanoid EP_2_ (EP_2_) receptor and large-conductance Ca^2+^-activated K^+^ (BK_Ca_) channels, respectively.

In the present study, we found that intravitreal injection of NOR3 induced retinal arteriolar dilation in rats and that this response was inhibited by a cyclooxygenase inhibitor or a prostanoid EP_2_ receptor blocker, but not by a prostanoid IP receptor blocker. These results suggest that the prostanoid EP_2_ receptor is involved in the glial cell-stimulated retinal vasodilation response (Figure 6).

Previously, we found that NMDA-induced neuron-derived NO acts on ryanodine receptors, increases Ca^2+^ concentrations in glial cells, and evokes dilation responses via EET and PG release in the rat retina [5]. Retinal nerve activity induced by light stimulation increases Ca^2+^ concentration in glial cells [10]. Light stimulation induces the release of EETs, PGs, and arachidonic acid cascade metabolites from glial cells, causing retinal blood vessels to dilate or constrict [11,12]. These results were consistent with those of our previous study. Although PGE_2_ has been suggested to be the PG released from glial cells in the brain and retina, other PGs may be involved, because these studies used cyclooxygenase inhibitors [12,13]. We have previously reported that PGE_2_ dilates retinal arterioles and that stimulation of the prostanoid EP_2_ receptor markedly dilates retinal arterioles, whereas stimulation of the prostanoid EP_4_ receptor has a much weaker vasodilatory effect [7]. We have also found that i.v. administration of NO donors dilates retinal arterioles not through the guanylyl cyclase-cGMP pathway but through the release of PGI_2_ via activation of cyclooxygenase in the retina [8,9]. These findings indicate that PGE_2_ and PGI_2_ are important factors in the regulation of retinal vascular tone. In the present study, we investigated the PGs involved in glial cell-stimulated retinal vasodilation in rats and showed, for the first time, that the PG released from glial cells in rats is PGE_2_, which acts on the prostanoid EP_2_ receptors. Based on our previous and present results, we propose that PGE_2_ and PGI_2_ are important for glial cell-mediated and vascular endothelial cell-mediated vasodilation in the retina, respectively.

In the present study, the retinal vasodilator response induced by intravitreal injection of NOR3 was not completely attenuated by the cyclooxygenase inhibitor. EETs are arachidonic acid cascade metabolites different to PGs and may be involved in the remaining NO donor-induced retinal vasodilator response in the presence of cyclooxygenase inhibitors (Figure 6), as we have reported previously [5]. In the previous report, we demonstrated that intravitreal injection of the NO donor is suppressed by approximately 60% by the structural analog of EET that inhibits their action [5]. In rat retinal tissue, guanylyl cyclase is expressed in the neuronal cell layer of the inner retina, whereas its expression in the retinal blood vessels is low [8]. In addition, the retinal vasodilator response to i.v. injection of the cGMP derivative is very weak [8]. Thus, cGMP produced in the retinal neurons by the intravitreal NO donor-activated guanylyl cyclase is not likely to dilate the retinal blood vessels. We propose that the guanylyl cyclase-cGMP pathway is not likely to be involved in retinal vasodilation by intravitreal injection of NO donors.

In rat models of glaucoma, the NMDA-induced retinal vasodilatory response is attenuated because neurons in the inner retinal layers are injured and degenerated [5]. In the retinas of diabetic rats, the vasodilatory responses mediated by neurons and glial cells are also attenuated, and impairment of large-conductance Ca^2+^-activated K^+^ channels, the point of action of EETs released from glial cells [14], is involved in the underlying mechanisms. Furthermore, several morphological or functional alterations in retinal neurons and glial cells have been reported in models of glaucoma and diabetes [3,15]. Therefore, in the pathogenesis of glaucoma and diabetes, neurovascular unit disruption may occur and alter the neuronal and glial cell-mediated regulation of vascular tone in the retina. However, the details of the mechanisms of disruption during the pathogenesis are still not clarified sufficiently. In addition, it remains unclear what kind of retinal glial cells, such as astrocytes and/or Müller cells, act on NO-induced retinal vasodilation. Further studies are required to clarify these issues.

In the brain, astrocytes modulate the redox homeostasis of the blood–brain barrier and regulate cerebral blood flow [16]. Oxidative stress and inflammation contribute to cerebral endothelial dysfunction and increased permeability of the blood–brain barrier. Direct damage to endothelial cells and the blood–brain barrier affects other components in the neurovascular unit, resulting in a further vicious cycle [16,17]. In the retina, as in the brain, oxidative stress-induced disruption of the neurovascular unit may lead to neuroinflammation and neurodegeneration. In fact, inflammation and oxidative stress have been reported to be involved in the development and progression of glaucoma and in the vascular lesions of diabetic retinopathy [18,19,20,21]. PGE_2_ affects inflammation, immune, and oxidative stress responses [22]. Moreover, PGE_2_ has been reported to be increased in patients with proliferative diabetic retinopathy [23]. These reports suggest that glaucoma and diabetes may adversely affect the neurovascular unit in the retina by altering PGE_2_ production. In contrast, PGE_2_ or stimulation of prostanoid EP_2_ receptors may have positive effects on the retina, such as inhibition of angiogenesis, decrease in intraocular pressure, and protection against retinal neuronal injury [24,25,26]. Therefore, further studies are needed to determine how PGE_2_ affects retinal vascular reactivity in pathological conditions. Transcriptome analysis, carried out using cerebral capillaries [27], may also be useful in retinal blood vessels to identify novel and highly selective therapeutic targets for various ocular diseases.

## 5. Conclusions

In summary, the present study demonstrates that the prostanoid EP_2_ receptor is required for NO-induced glial cell-mediated vasodilation in the rat retina. Glial cell-derived PGE_2_ and EETs may play essential roles in retinal vasodilation.

## Figures and Tables

**Table 1:** The baseline retinal arteriolar diameter, mean arterial pressure, and heart rate values.

Treatments	Retinal Arteriolar Diameter (µm)	Mean Arterial Pressure (mmHg)	Heart Rate (Beats/min)
Protocol 1			
Vehicle (*n* = 5)	44 ± 2	112 ± 2	371 ± 17
Indomethacin (*n* = 5)	45 ± 2	112 ± 1	371 ± 3
Protocol 2			
Vehicle (*n* = 8)	44 ± 3	110 ± 2	399 ± 8
PF-04418948 (*n* = 9)	46 ± 3	113 ± 2	381 ± 8
Protocol 3			
Vehicle (*n* = 8)	43 ± 2	111 ± 2	379 ± 8
CAY10441 (*n* = 9)	46 ± 1	112 ± 1	372 ± 10

Values are expressed as mean ± S.E.M. These values were measured just before injection or infusion of vasodilators.

## Data Availability

The data presented in this study are available upon request from the corresponding author.
